# The impact of driving versus undistracted listening on podcast knowledge acquisition and retention using a driving simulator: A randomized, cross-over trial

**DOI:** 10.1371/journal.pone.0331299

**Published:** 2025-09-08

**Authors:** Yasaman Jabbari, Michael Gottlieb, Martin von Mohrenschildt, Mark Lee, Kristen Arnold, Judith M. Shedden, Jonathan Sherbino

**Affiliations:** 1 Department of Psychology, Neuroscience & Behaviour, McMaster University, Hamilton, Ontario, Canada; 2 Department of Emergency Medicine, Rush University Medical Center, Chicago, Illinois, United States of America; 3 Faculty of Engineering, McMaster University, Hamilton, Ontario, Canada; 4 McMaster Education Research, Innovation & Theory (MERIT) Program, McMaster University, Hamilton, Ontario, Canada; 5 Department of Medicine, McMaster University, Hamilton, Ontario, Canada; Islamic Azad University Ahvaz Branch, IRAN, ISLAMIC REPUBLIC OF

## Abstract

**Introduction:**

Research on listening to podcasts while driving suggested no significant difference compared to undistracted listening. However, these studies were conducted in non-controlled driving environments, limiting the evaluation of the environment’s impact. This study aimed to compare knowledge acquisition and retention among resident physicians and undergraduate students while listening to medical education podcasts in a controlled, simulator-based, driving environment versus an undistracted listening condition.

**Methods:**

A randomized, crossover trial involved 19 residents and 22 undergraduate students from McMaster university and McMaster hospital. Participants listened to podcasts while driving in an immersive, high-fidelity motion simulator that mimics different driving environments: a high-distraction city environment and a low-distraction country environment. In the undistracted listening condition, participants listened to podcasts while being seated at a desk. Immediate and delayed recall tests after a month were administered, and data were analyzed using a 2x2 mixed ANOVA.

**Results:**

There were no significant differences in knowledge acquisition (e.g., accuracy) between the undistracted, city driving, and country driving conditions (*p* > 0.05, *η*2 = 0.011). However, the country driving condition demonstrated slightly higher accuracy compared to the city driving condition in the immediate assessment condition (*p* < 0.05, *η*2 = 0.018). Medical expertise level (i.e., resident vs student) did not affect knowledge acquisition across different listening conditions (*p* > 0.05, *η*2 = 0.007).

**Conclusion:**

In a simulated environment, knowledge acquisition from a podcast is not compromised by the attention needed for driving a vehicle. The two distraction levels used in this experiment showed no significant interference with knowledge acquisition. This holds true regardless of participants’ medical expertise. This study highlights the potential of incorporating podcasts into daily commuting to support ongoing education without requiring dedicated study time, enhancing both flexibility and efficiency in professional development.

## Introduction

In today’s fast-paced world, podcasts have become increasingly popular as a convenient and accessible medium for learning, especially among healthcare professionals [11,12,18,20]. With hectic schedules and limited time for dedicated study, many medical residents turn to podcasts as a means of acquiring knowledge and staying informed. The flexibility of podcasts allows for learning opportunities in various settings, such as driving, doing chores, and exercising. Among these activities, driving has emerged as the most favorable simultaneous activity for podcast listening, enabling individuals to make productive use of their travel time [6, 14]. However, the question of how this multitasking approach impacts knowledge acquisition and retention during podcast consumption remains underexplored.

Our previous study [5] revealed no significant differences in learning outcomes when comparing medical residents listening to a podcast in an undistracted environment versus listening to a podcast during a routine commute by car. However, this study was limited by the lack of a controlled test setting; each resident used a personalized, real-world commute, where some features of driving were likely automatized. In contrast, the current study incorporates a controlled setting, utilizing a driving motion simulator to simulate real-world driving scenarios such as country or city environments. Automatization of the driving task was not possible, as all routes were new to participants. Simulators offer a safe, affordable, and realistic environment that closely mirrors real-world driving settings, making them highly suitable for testing driving behavior [2,8–10,13]. To the best of our knowledge, this study is among the first to assess knowledge acquisition from listening to medical educational podcasts while driving, using a driving simulator. This underscores the significance of our study’s aims to provide reliable data within a controlled yet realistic driving setup.

Specifically, this study examines how listening to medical educational podcasts in different driving conditions (city versus country) affects knowledge acquisition compared to an undistracted setting. The study is grounded in threaded cognition theory [21], which suggests that tasks relying on separate cognitive resources, such as auditory processing for podcast learning and visuospatial processing for driving can occur simultaneously without interference. Additionally, the modal model of memory [1] posits that memory is processed in distinct pathways within working memory. These frameworks suggest that podcast-based learning should be possible while driving, provided that cognitive demands remain manageable. By applying these theories, this study investigates whether driving conditions affect knowledge acquisition.

Additionally, the current study aims to investigate the role of medical expertise in podcast learning by comparing non-medical students with no experience to experienced healthcare providers to assess whether prior domain knowledge influences learning outcomes in multitasking environments.

This study provides valuable insights into the potential for podcasts as an educational tool during everyday activities, particularly in high-demand professions. By assessing knowledge acquisition in a realistic driving simulation, it advances our understanding of effective multitasking in professional learning environments.

## Method

### Study setting

This study was a randomized, crossover trial comparing knowledge acquisition and retention (immediate and delayed recall) from medical education podcasts under different learning conditions. Participants listened to podcasts while driving in high distraction (e.g., city versus low distraction (e.g., country) environments compared with an undistracted listening condition while seated at a desk in a quiet location. We recruited undergraduate students from McMaster University through SONA, the undergraduate research pool, while resident physicians from Emergency Medicine, Internal Medicine, and Anesthesiology were recruited through internal announcements within their respective training programs (Table 1). Inclusion criteria consisted of participants who had a valid driving license and no reported sensitivity to motion sickness, vertigo, or claustrophobia. Participants were compensated for their time with residents receiving gift cards for both immediate and delayed assessments ($75 total), while undergraduate students received 1.5 course credits for the immediate assessment and $25 total gift cards for the delayed assessment. Prior to beginning the experiment, each participant provided written, informed consent. The recruitment period for the study began on August 17th, 2022, and concluded on March 31st 2023. Ethical clearance for this study was granted by the Hamilton Integrated Research Ethics Board (HiREB Project ID: 7310).

**Table 1:** Demographic information of participants.

Participant Group	Sample Size	Specialty	Program Level	Podcast Listening Frequency
Residents	n = 19	Emergency Medicine, Internal Medicine, Anesthesiology	9 Junior Residents (PGY-1 and PGY-2) 10 Senior Residents (PGY-3 and PGY-4)	At least once per day: 19
Undergraduate, non-medical students	n = 22	N/A	17 Junior Students (Level-1) 5 Senior Students (Level 3, 4 and 5)	At least once per day: 0 At least once per week: 3 At least once per month: 9 At least once per 6 month: 3 Never: 7

### Study design

Counterbalanced randomization of podcast episodes and experimental conditions was used. The study consisted of two components: an in-person educational intervention and an online assessment. The in-person component involved four steps, including listening to two different podcast episodes and two related knowledge recall tests. The podcast listening sessions included two experimental conditions: driving and undistracted. In the driving condition, participants listened to a podcast while driving in a virtual environment that simulated either city roads (high cognitive load) or country roads (low cognitive load). In the undistracted listening condition, participants listened to a different podcast while seated at a desk. Following each condition, participants completed a knowledge acquisition test that was mapped to the specific podcast. To assess long term knowledge retention, participants completed an online, new knowledge assessment, at one month. The tests were administered through LimeSurvey.

### Materials and stimuli-virtual environment

**Fig 1:**
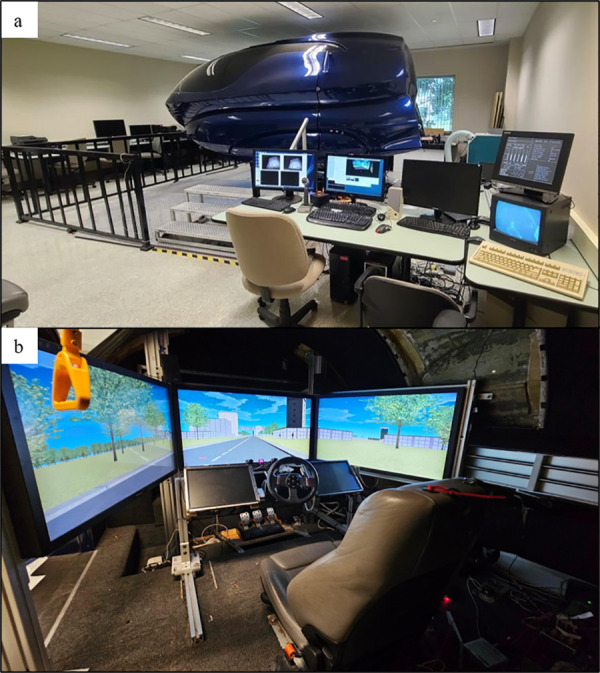
image “a”, represents the outside of the simulator and the control interface and image “b” represents the inside of the simulator and the location of various controls, such as LCDs, steering wheel, gas pedal, and brake pedal.

**Fig 2:**
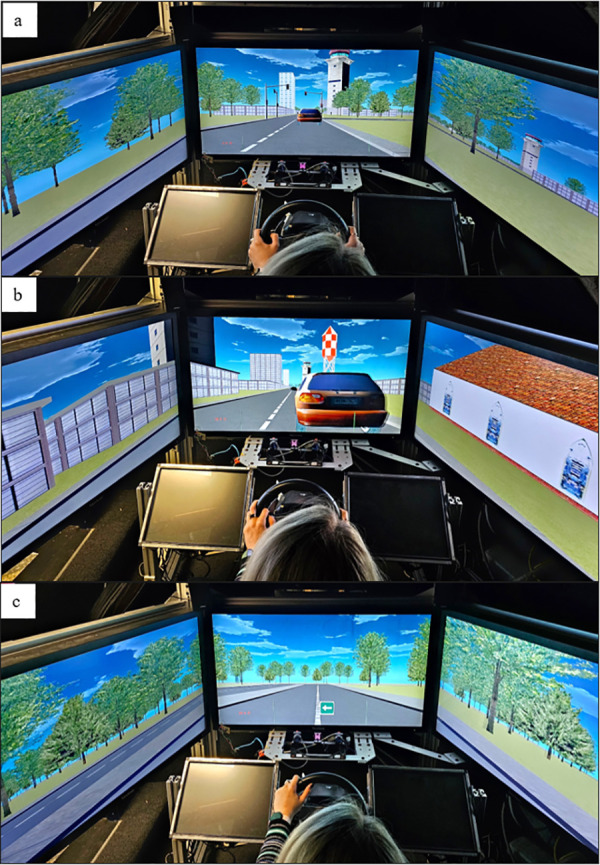
image “a” and “b”, represent a simulated environment that illustrates city roads, while image “c” represents country roads. During driving conditions (city and country), a green and white guiding arrow appears in the middle of the screen to indicate upcoming turns, as shown in the bottom image.

The experiment employed a virtual town driving simulation, displayed across three LCD screens arranged to offer a 120° horizontal field of view. Participants used a Logitech steering wheel and pedals for interaction. An intercom system facilitated communication between participants and experimenters (Fig 1). To simulate various possible real-world scenarios of podcast listening while driving, we designed virtual environments with two levels of cognitive demand. The city environment reproduced a high cognitive demand context that required drivers’ constant attention on the road. This design aimed to replicate a large city with multiple roadways with moving vehicles and intersections, creating different city blocks. The sidewalks had various pedestrians passing by occasionally, and landmarks such as shops, a church, high rises, and houses along the routes, giving the impression of a busy city. Additionally, multiple traffic lights regulated traffic flow, and drivers were encouraged to obey traffic rules. The country environment reproduced a low cognitive demand context that required less attention on the road. This design consisted of long rural roads without traffic lights, other vehicles, and pedestrians (Fig 2). Out of the six podcast segments assigned to the driving condition, three podcasts were listened to while driving in the city condition and the other three in country condition, with the order of presentation of the podcasts being counterbalanced.

During the driving condition, participants were guided along various routes, with each route beginning at a different location in the virtual environments. The routes featured multiple turning decision points (e.g., intersections) and as the car approached a decision point, a guiding arrow was overlaid on the screen to indicate whether a left or right turn was required. Participants were instructed to follow standard residential area road rules, such as driving in the right lane at 30–40 km/h. At the end of each podcast segment, the driving task was paused, and participants initiated the next podcast segment by pushing the start lever located behind the steering wheel.

### Podcast creation

Each 30-minute podcast included a two-person discussion of six medical journal articles. The selected podcast segments were used (with permission) from an originally produced medical education podcast. The articles and their corresponding podcast segments were screened by study investigators to ensure that they were not previously discussed in any of the respective residency programs’ curricula or journal clubs. Podcasts were excluded if they were syndicated on major podcast programs or discussed on social media (defined as appearing within the first 10 results on www.googlefoam.com).

### Question development

The research team created two tests for immediate recall, each consisting of 61 and 63 items (approximately 10 questions per podcast segment), and one 40-item test for delayed recall. The tests were developed following best practices in test-item creation [3], using multiple-choice questions with four alphabetically listed options, one of which was the single best answer. The questions for the tests were reviewed by experienced test item writers who provided feedback on wording, difficulty, and format [5,4]. The internal consistency of each test was established using Cronbach’s alpha [25], and item analysis was conducted using two examination performance measures: item difficulty and discrimination index. The final Cronbach’s alpha for the two test versions was 0.70 and 0.71, with test difficulty set at approximately 0.76 for each version. The tests were matched for reliability and difficulty.

### Statistical analysis

Recall score was analyzed using a 2x2 mixed-design analysis of variance (ANOVA) with Greenhouse-Geisser sphericity correction. Analysis was conducted using Jamovi statistical software [19,26]. The design incorporated the dependent variable of “recall score” and two within-subject independent variables: (1) the test administration time (“Time”: immediate versus delayed) and (2) podcast listening conditions (“Condition”: country driving versus city driving versus undistracted). A between-subject independent variable compared two participant groups which differed in podcast content expertise (“Expertise”: medical residents versus non-medical undergraduate students). Further exploration of significant results was achieved through post hoc analyses using Tukey’s HSD test.

## Results

We recruited 19 medical residents and 22 non-medical undergraduate students for the study. All participants took part in both immediate and delayed assessments, with the exception of two individuals, one medical resident and one non-medical student, who did not complete the delayed assessment. The main effect of podcast listening conditions was not statistically significant, suggesting that neither immediate nor delayed recall score was significantly different among the undistracted, country driving, and city driving conditions (*F* (1.83, 67.55) = 3.110, Msq = 0.053, *p* > 0.05, *η*^*2*^ = 0.011). See Table 2 for further details.

**Table 2:** Mean proportion and the standard deviation of the correct recall score for “participants group” (residents and undergraduates), separated by podcast listening “condition” (drive (city and country) and undistracted) and test administration “time” (delayed and immediate).

Participants Group	Time	Condition	Mean	SD
*Residents*				
	*Immediate* *n = 19*	*Driving*	0.675	0.077
		* City*	0.666	0.083
		* Country*	0.682	0.115
		*Undistracted*	0.709	0.105
	*Delayed* *n = 18*	*Driving*	0.625	0.142
		* City*	0.629	0.164
		* Country*	0.639	0.177
		*Undistracted*	0.581	0.114
*Undergraduates*				
	*Immediate* *n = 22*	*Driving*	0.439	0.123
		* City*	0.382	0.126
		* Country*	0.495	0.167
		*Undistracted*	0.551	0.154
	*Delayed* *n = 21*	*Driving*	0.355	0.092
		* City*	0.349	0.165
		* Country*	0.359	0.173
		*Undistracted*	0.362	0.097

Subanalysis indicated a small but statistically significant interaction between listening condition and assessment time (*F* (1.83, 68.83) = 4.498, Msq = 0.085, *p* < 0.05, *η*^*2*^ = 0.018). Participants demonstrated better immediate recall in a lower cognitive load driving context (e.g., country) than high cognitive load context (e.g., city). (*t* (37) = − 3.014, *p <* 0.05). This difference was no*t* observed in the delayed assessment.

**Fig 3:**
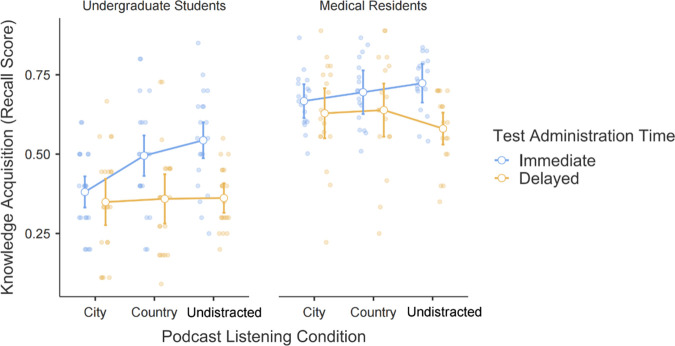
A plot illustrating the results of the 2x2 mixed ANOVA conducted to evaluate the influence of various podcast listening conditions (city, country, and undistracted) and test administration times (immediate and delayed) on the knowledge acquisition, which indicates the proportion correct of recall score of residents and undergraduates. The error bars represent 95% confidence intervals.

The main effect of medical expertise level was statistically significant, suggesting there was a significant difference in recall score between medical residents and non-medical students (*F* (1, 37) = 101.76, Msq = 3.367, *p* < 0.0001, *η*^*2*^ = 0.39). Medical residents had higher recall scores (*t* (37) = 10.08, *p* < .0001). However, *t*here was no statistically significant interaction between medical expertise and assessment time (*F* (1, 37) = 1.057, Msq = 0.020, *p* > 0.05, *η*^*2*^ = 0.002), nor podcast listening condition (*F* (1.83, 67.55) = 2.170, Msq = 0.037, *p* > 0.05, *η*^*2*^ = 0.007), nor a three-way interaction between medical expertise level, time, and condition (*F* (1.86, 68.83) = 0.519, Msq = 0.010, *p* > 0.05, *η*^*2*^ = 0.002), (Fig 3).

As anticipated, there was a decay in knowledge retention between immediate and delayed testing (*F* (1, 37) = 29.09, Msq = 0.556, *p* < 0.0001, *η*^*2*^ = 0.06). Immediate assessment had a higher recall score than delayed (*t* (37) = 5.394, *p* < .0001).

## Discussion

This study explored and offered insights into the feasibility of podcast-based learning in multitasking environments, particularly for busy professionals. In today’s productivity-driven world, this research highlights the potential of educational podcasts as a flexible and efficient tool for learning during routine activities like driving.

The present study built upon our previous research, investigating the impact of listening conditions (driving versus undistracted) on knowledge acquisition and retention from educational podcasts [5]. The limitations of the previous study included a lack of control over the conditions, as participants were self-reporting their driving leading to less experimental control of the driving condition. For instance, participants may have opted for familiar everyday routes, resulting in a low cognitive load driving condition. To address these limitations, we incorporated a controlled laboratory setting using a motion simulator. This enabled us to replicate various driving environments with different levels of cognitive demands, ensuring a more controlled and rigorous investigation into the impact of listening conditions on knowledge acquisition and retention from educational podcasts among medical residents. Additionally, we compared resident physicians, who were experienced with podcasts and had a high level of expertise in the materials presented, to undergraduate students with limited exposure to podcasts and no medical expertise.

Overall, the different listening conditions did not lead to noticeable differences in participants’ recall performance, in line with Gottlieb and colleagues [5], indicating that listening to podcasts while driving does not negatively affect knowledge acquisition compared to undistracted listening. Our findings provide support for the theory of threaded cognition, which suggests humans can engage in multitasking effectively by allocating different ‘threads’ of cognitive resources to each task. This theory clarifies that when tasks involve disparate cognitive processes (e.g., visual versus auditory), they can proceed concurrently with minimal interference [21]. Thus, our study aligns driving, a task demanding visual cognitive resources, with podcast listening, which uses auditory resources, to demonstrate that multitasking in such scenarios does not significantly hinder knowledge acquisition or retention.

When tasks involve little overlap in mental processing, multiple threads can run independently without interference [22]. However, when two tasks require the same cognitive resource simultaneously, performance is typically reduced [15,17,21,24]. These results are also consistent with the modal model of memory, which proposes discrete pathways within working memory [1,7]. In light of these compelling findings, it becomes evident that leveraging the theory of threaded cognition opens up exciting possibilities for optimizing learning experiences in multitasking scenarios. By recognizing the unique cognitive demands of different tasks, we can design educational interventions that maximize knowledge acquisition and retention, fostering a more efficient and seamless learning process.

We also observed some interesting interactions. In the immediate assessment, demanding city driving resulted in lower recall accuracy than the less demanding country environment, attributed to participants’ diverted attention. These results align with cognitive load theory, which suggests the high cognitive demands of city driving likely interfered with encoding, leading to lower immediate recall accuracy. However, this effect did not persist in the delayed assessment, suggesting that once information was stored in long-term memory, retrieval was less affected by prior encoding conditions [16,23]. This pattern is also supported by the modal model of memory [1], which differentiates between short-term memory, which is vulnerable to interference, and long-term memory, where consolidation ensures stability. These findings reinforce that while a high cognitive load may impair immediate learning outcomes, long-term retention remains resilient once encoding is successful.

The accuracy of the medical residents in the assessments was higher than that of the undergraduate students, which is not surprising given their greater familiarity with the educational material. Interestingly, even among undergraduate students, for whom a medical podcast might be cognitively demanding, the additional task of driving did not worsen their knowledge acquisition scores. This suggests that the cognitive load required for driving does not negatively influence the effectiveness of podcast-based learning, even when the content is specialized or challenging. These findings reinforce the notion that listening to podcasts while driving can be done effectively without compromising learning outcomes.

### Practical recommendations for educators

Our findings suggest that educational podcasts can be effectively integrated into multitasking environments without significant impairment to learning outcomes. While high cognitive load conditions, such as city driving, may momentarily reduce immediate recall, long-term retention remains unaffected. This reinforces the idea that podcast-based learning can be a productive strategy, even in demanding environments. Rather than discouraging multitasking, educators should focus on structuring content for clarity and engagement, ensuring that material is well-organized and easy to follow. Since prior knowledge facilitates learning, novice learners may benefit from simplified explanations and guided reflection, while experienced learners can engage with more complex discussions that build on their existing knowledge. Given that immediate recall may be slightly affected by cognitive load, reinforcing key concepts through structured review methods, such as periodic recaps and discussion prompts, may help enhance retention. These findings support the integration of podcasts into professional and academic learning without the need for rigid constraints on listening conditions. Multitasking is a viable and efficient approach, allowing learners to make productive use of time without compromising knowledge retention.

### Limitations and future directions

While the experimental design was improved, it’s important to note that this study is an extension of [5] and thus centers around driving as the multitasking activity. While other research has demonstrated a similar finding with exercise [4], subsequent research endeavors should delve into the impacts of podcast listening during various other activities to achieve a more holistic comprehension. Even though participants in this study encountered unfamiliar roads, it’s important to highlight that we integrated GPS guidance for wayfinding. A potential avenue for future exploration could involve enhancing the cognitive demand of the task by introducing spatial cognitive challenges through wayfinding, without relying on GPS assistance. However, such a scenario might be relatively uncommon due to the prevalent use of GPS in everyday driving.

Additionally, while this study compared medical residents and undergraduate students to examine the role of expertise in podcast-based learning, the sample size may not have been sufficient to detect more subtle differences. While the reported small effect sizes might suggest limited practical significance, even minor improvements in learning efficiency can be valuable, particularly in real-world educational settings where cumulative learning experiences over time could lead to meaningful gains. Future research should aim for larger and more diverse samples to improve statistical power and further investigate how expertise influences learning outcomes in multitasking environments. Additionally, aggregating data across similar studies or conducting meta-analyses could help clarify small but meaningful effects that may not be observable in single studies.

Another limitation of this study is the lack of demographic data, which restricted our ability to examine potential moderating effects of individual differences such as age, gender, prior experience with multitasking, cognitive capacity, or learning preferences. Future studies should incorporate detailed participant characteristics to assess their influence on learning outcomes. Expanding research to other multitasking contexts, such as navigation without GPS assistance, may also provide a more comprehensive understanding of how cognitive load impacts podcast-based learning.

## Conclusions

This study underscores the significance of aligning educational strategies with the cognitive realities of multitasking, particularly in contexts like driving. It equips educators with insights to refine teaching methods that complement podcast learning during such activities. Simultaneously, it guides learners towards more strategic podcast consumption, enhancing learning efficiency across varied subjects. These practical applications signify a step forward in optimizing educational practices for multitasking environments. With these advancements in mind, we can continue to explore innovative ways to integrate learning with our everyday activities, enhancing the efficiency and effectiveness of education in an increasingly dynamic world.

## Supporting information

S1 Appendix Additional sensitivity analysis on driving speed and podcast listening frequency. This appendix includes two analyses: (1) a Pearson correlation between driving speed and recall accuracy in city and country driving conditions, and (2) an analysis of self-reported podcast listening frequency in relation to immediate and delayed assessment performance.
(DOCX)
