# Supplementary material for: The impact of driving versus undistracted listening on podcast knowledge acquisition and retention using a driving simulator: A randomized, cross-over trial
Source: PLoS One. 2025 Sep 8;20(9):e0331299. doi: 10.1371/journal.pone.0331299 (PMC12416652; doi:10.1371/journal.pone.0331299)
Supplement: S1 Appendix — This appendix includes two analyses: (1) a Pearson correlation between driving speed and recall accuracy in city and country driving conditions, and (2) an analysis of self-reported podcast listening frequency in relation to immediate and delayed assessment performance. (DOCX) [file pone.0331299.s001.docx]

# S1 Appendix. Additional sensitivity analysis on driving speed and podcast listening frequency.

The appendix includes an additional sensitivity analysis that assesses driving speed, and self-reported frequency of podcast listening.

## *Driving Speed*

A Pearson correlation analysis was performed to explore the potential relationship between driving speed and accuracy on the recall assessments among participants after the driving condition. The results showed no statistically significant association between driving speed and accuracy in both the city and country conditions. This lack of correlation might be attributed to the instructions given to participants, encouraging them to adhere to traffic rules and drive as they would in real-world scenarios, with a speed range of 40-50 km/h.

## *Self-reported Frequency of Podcast Listening*

The frequency of podcast listening was reported on a scale from once per day to never by both undergraduates and residents. While all residents reported listening to podcasts every day, the frequency varied among undergraduates (Table 1). In our analysis, we explored the relationship between self-reported podcast listening frequency and accuracy in immediate and delayed assessments for undergraduates across the undistracted, city, and country conditions. A Pearson correlation revealed a significant positive association, indicating that undergraduates who reported a higher frequency of podcast listening achieved higher scores in the immediate assessment of the undistracted condition (*r* (22) = 0.489, *p* = 0.021). This finding suggests that expertise and familiarity with podcast consumption may contribute to improved performance.
